# Electroretinography in the Collared Scops Owl (*Otus lettia*)

**DOI:** 10.3390/vetsci13060570

**Published:** 2026-06-10

**Authors:** Yun-Shan Chiu, Chau-Hwa Chie, Carmen Colitz, Pin-Huan Yu, I-Han Wu, Chung-Tien Lin

**Affiliations:** 1 Institute of Veterinary Clinical Sciences, School of Veterinary Medicine, National Taiwan University, No. 153, Sec. 3, Keelung Rd., Da-an Dist., Taipei City 10672, Taiwan; greenshanshan@gmail.com (Y.-S.C.); chie@ntu.edu.tw (C.-H.C.); pinhuan@ntu.edu.tw (P.-H.Y.); 2 MedVet Jupiter Emergency Vet & Specialty Care, 505 Commerce Way, Jupiter, FL 33458, USA; ccolitz@gmail.com; 3 iHorizon Veterinary Hospital, No. 436, Dongqiao 3rd Rd., Yongkang Dist., Tainan City 710038, Taiwan; yihanwu59@gmail.com; 4 Department of Ophthalmology, National Taiwan University Veterinary Hospital, No. 153, Sec. 3, Keelung Rd., Da-an Dist., Taipei City 10672, Taiwan

**Keywords:** Collared scops owl, electroretinography, *Otus lettia*, raptor, retina

## Abstract

Electroretinography (ERG) is a non-invasive technique used to assess retinal function via electrical responses to light stimuli. We established baseline ERG parameters and a standardized recording protocol for collared scops owls (*Otus lettia*). Twelve eyes of six owls were evaluated. In addition to the pre-release assessment, ocular reflex tests and basic ophthalmic examinations were performed before the induction of anesthesia. Routine radiographic and hematological examinations were performed under general anesthesia, followed by ERG recordings. Under scotopic –20 dB conditions, the a-wave amplitude was 1.78 ± 0.53 μV (implicit time: 37.83 ± 5.52 ms), and the b-wave was 41.59 ± 10.71 μV (100.88 ± 10.9 ms). For scotopic 0 dB mixed responses, the a-wave amplitude was 27.98 ± 5.9 μV (27.64 ± 2.71 ms), and that of the b-wave was 175.51 ± 13.82 μV (97.02 ± 7.01 ms). Under photopic conditions, the a-wave and b-wave amplitudes were 2.88 ± 2.06 μV (28.67 ± 2.77 ms) and 25.53 ± 10.61 μV (77.78 ± 16.18 ms). To the best of our knowledge, this is the first study to establish species-specific baseline ERG parameters for collared scops owls. These findings provide a valuable tool for assessing retinal function in raptors and may serve as a baseline framework for ERG evaluation in other avian species.

## Simple Summary

The retina is a light-sensitive tissue in the eye that plays a key role in vision. Electroretinography is a non-invasive test used to measure how the retina responds to light. However, normal reference values for this test are not available for many bird species, including the collared scops owl. In this study, six owls were examined, and their retinal responses were recorded under different light conditions using a standardized method. The results provide the first baseline data for retinal function in this species. These findings can help veterinarians better assess eye health, detect retinal diseases, and guide treatment decisions. This study may also serve as a useful reference for future research and clinical use in other bird species.

## 1. Introduction

Large eyes relative to body size in raptors are an anatomical adaptation that enhances visual sensitivity and acuity and facilitates survival in nature [1]. However, this adaptation also increases their susceptibility to ocular injuries, including trauma affecting the anterior and posterior segments, particularly the uvea and retina [2,3]. Cataracts are another recognized cause of visual impairment in birds of prey and primarily affect vision through lens opacity rather than through direct retinal dysfunction [4,5]. Visual impairment resulting from retinal injury or cataract formation can significantly compromise the quality of life and reduce the likelihood of successful reintroduction into the wild [6]. Therefore, objective evaluation of retinal function in live raptors is crucial for assessing visual capability.

Flash electroretinography (ERG) is a non-invasive electrodiagnostic test that represents the electrical mass response of the retina to light stimulation [7]. It is widely utilized in veterinary ophthalmology and serves multiple purposes, including preoperative retinal function assessment before cataract surgery when fundic examination is unfeasible, evaluation of visual loss in cases where retinal lesions are undetectable via ophthalmoscopy, and diagnosis of inherited retinal disorders [7,8]. Furthermore, ERG serves as a crucial tool in ophthalmological research, as well as in pharmaceutical and toxicological screening for the adverse effects of drugs and other chemical compounds [9].

Species-specific ERG characteristics have been reported in several avian species, including parrots and certain raptors [10,11,12]. ERG findings should be interpreted using data obtained from validated protocols for the species under investigation because of the considerable interspecies variation in the retinal structure among birds [13].

The collared scops owl (*Otus lettia*) is a small nocturnal raptor commonly found in Taiwan that primarily inhabits low-altitude hilly regions across the island. This species frequently resides near human settlements and is recognized for its high frequency of human interactions in Taiwan [14]. Owing to this close association, it is one of the most frequently treated species in wildlife rescue and rehabilitation centers [15].

Baseline electroretinographic latencies and amplitudes in the collared scops owls were characterized. Baseline flash ERG data obtained using a standardized protocol may serve as a complementary tool for objectively assessing retinal function, particularly in individuals with suspected retinal involvement associated with ocular injury or disease, as well as for preoperative retinal evaluation prior to cataract surgery. The data presented in this study provide foundational baseline information for future clinical research involving raptors.

## 2. Materials and Methods

This study was approved by the Institutional Animal Care and Use Committee (IACUC) of National Taiwan University (approval number: NTU-114-EL-00071).

Free-living collared scops owls (*O. lettia*) admitted to a certified Wildlife Rescue Center in Taiwan between July 2025 and November 2025 were eligible for enrollment. All individuals were rescued due to different traumatic conditions, including head trauma, juvenile falls from the nest, and limb fractures, and were managed under standard rehabilitation protocols with the primary aim of clinical stabilization and eventual release.

Upon admission, each owl underwent a complete physical and clinical assessment, and appropriate medical or supportive treatment was provided as needed. Ophthalmic examinations were performed as part of the initial clinical evaluation and repeated before release.

General anesthesia was not specifically administered for this study. Instead, anesthesia was clinically indicated for routine pre-release procedures, including radiographic imaging and blood sampling. Ocular reflex and basic ophthalmic examinations were performed before anesthesia. ERG recordings were subsequently performed during the same anesthetic event to avoid additional handling and minimize stress to the animals.

The age and sex of the owls were unknown; however, all individuals were classified as adults based on their plumage characteristics. Body weight, measured before rehydration and anesthesia, ranged from 150 g to 180 g.

Six owls were included in this study. Twelve eyes of these six individuals were examined and selected for analysis after no abnormalities were detected on ophthalmic examination and pre-release assessment. Ocular evaluation included the assessment of the palpebral reflex, dazzle reflex, direct pupillary light reflexes, fluorescein staining (Omni Fluro; Opto Hellas, Katerini, Greece), slit-lamp biomicroscopy (Kowa SL-17; Kowa, Nagoya, Japan), rebound tonometry (Tonovet; iCare, Vantaa, Finland), and indirect ophthalmoscopy using a 40-diopter condensing lens (40D lens; Volk Optical Inc., Mentor, OH, USA).

ERG was performed under general anesthesia. Anesthesia was induced using 5% isoflurane (Attane; Piramal Critical Care, Bethlehem, PA, USA) delivered via an induction chamber and maintained at 1.5–2% isoflurane following tracheal intubation. The heart rate, respiratory rate, body temperature, and depth of anesthesia were closely monitored throughout the procedure by a licensed veterinarian experienced in exotic animal anesthesia. After completing the ERG examination, gas anesthesia was discontinued, and the birds were extubated and placed in an oxygen-enriched chamber until they fully regained consciousness. The recovered owls were returned to their original enclosures on the same day and were prepared for release into the wild. The pupils were fully dilated throughout the examination despite the absence of mydriatic agents, and their sizes were evaluated at the beginning and end of the ERG recordings. An eyelid speculum (F1-101; Fine Science Tools, Foster City, CA, USA) was used to keep the eyes open.

ERG recordings were performed using a hand-held ERG machine (BPM-300 Electrodiagnostic System; RetinoGraphics, Newton, CT, USA) that incorporated an integrated full-color stimulator delivering diffuse, uniformly illuminated flashes. The stimulus provided a broad-spectrum light source with adjustable intensity and background illumination, rather than a single-wavelength source. According to the photometric calibration of the ERG system, a stimulus intensity of 0 dB corresponded to 3 cd/m^2^.

**Figure 1:**
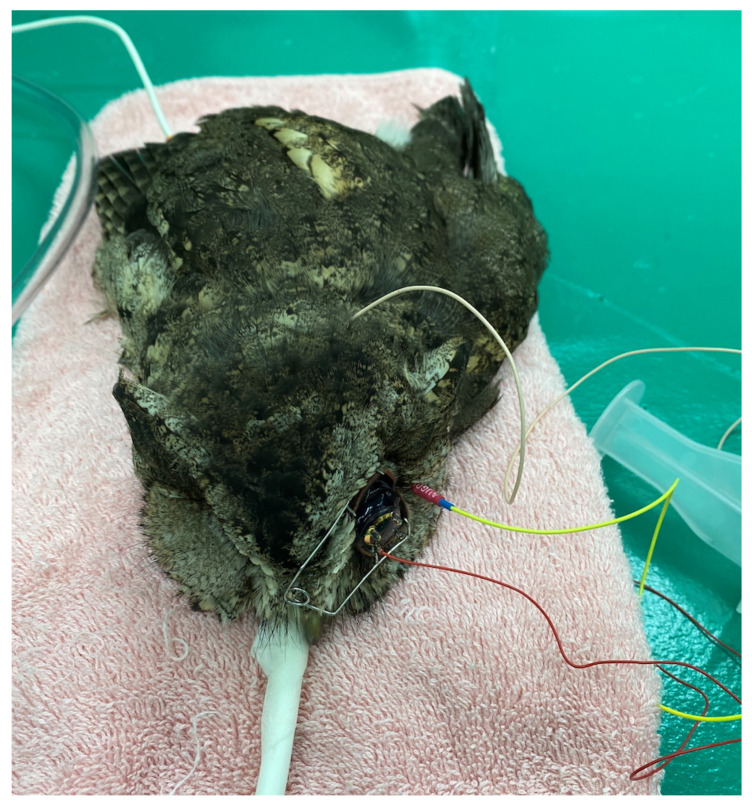
Electroretinographic (ERG) recording in an anesthetized collared scops owl (*Otus lettia*). A contact lens electrode was placed on the corneal surface, with gel-based artificial tears used as a stabilizing medium. An eyelid speculum was applied to keep the eyes open during the recording. The ground electrode (white wire) was inserted subcutaneously at the apex of the occiput, and the reference electrode (yellow wire) was positioned approximately 0.5–1 cm lateral to the lateral canthus of the tested eye.

A gold-plated monopolar contact lens electrode (ERG-jet; The Electrode Store, Enumclaw, WA, USA) was applied to the cornea using artificial tears (Optixcare Eye Lube Plus; Aventix Animal Health, ON, Canada) as the contact lens-stabilizing gel. Subdermal platinum-iridium needle electrodes (PLAT-5PK; The Electrode Store, Enumclaw, WA, USA) were used as the reference and ground electrodes, respectively. The ground electrode was inserted subcutaneously at the apex of the occiput, whereas the reference electrode was placed approximately 0.5–1 cm lateral to the lateral canthus of the tested eye (Figure 1). The light stimuli were delivered via a flash source positioned 5 cm from the test eye, perpendicular to the cornea. The band-pass filter was set to 0.4–400 Hz. Electrode impedance was maintained at or below 5 kΩ.

The ERG recordings were obtained from the eyes of each owl. Two ERG protocols were sequentially performed for each eye. One eye was randomly selected for initial testing, and the same procedure was subsequently applied to the contralateral eye. The ERG protocols were designed based on previously published studies on raptors [11] and adapted to align with the technical specifications of the BPM-300 system.

The first protocol was conducted under scotopic conditions after dark adaptation for 20 min. During the adaptation period, both eyes were kept dark. ERG responses were recorded to four low-intensity light flashes at −20 dB with 2-s interstimulus intervals, followed by two flashes at 0 dB and another two at +5 dB. Under these scotopic conditions, the initial low-intensity stimuli predominantly elicited rod-driven responses, whereas the responses to subsequent higher-intensity flashes (0 and +5 dB) reflected mixed activation of rod and cone photoreceptors. All procedures were conducted in a controlled darkened examination environment to minimize external light interference.

The second protocol was performed under photopic conditions to assess the cone-mediated responses. Following 10 min of light adaptation with white background light at an intensity of 30 cd/m^2^, both eyes were exposed to background light. ERG responses to 16 flashes at 0 dB were delivered at 2-s intervals and recorded and averaged to obtain the final waveform data. The background illumination was maintained throughout the recording period.

The amplitude of the a-wave was measured from the baseline to its trough, whereas that of the b-wave was measured from the a-wave trough to the b-wave peak. The implicit times of the a- and b-waves were determined as the intervals from stimulus onset to the a- and b-wave peaks, respectively.

Statistical analyses were performed using IBM SPSS Statistics (version 25.0; IBM Corp., Armonk, NY, USA). Data normality was assessed using the Shapiro–Wilk test. The eye was considered the unit of analysis, and electroretinographic data from both eyes of each owl were included in the analyses. Because the primary objective of this study was to establish preliminary baseline electroretinographic parameters for the collared scops owl, data from all examined eyes were summarized using descriptive statistics, including mean, standard deviation (SD), minimum, maximum, and 95% confidence intervals (CI).

## 3. Results

**Figure 2:**
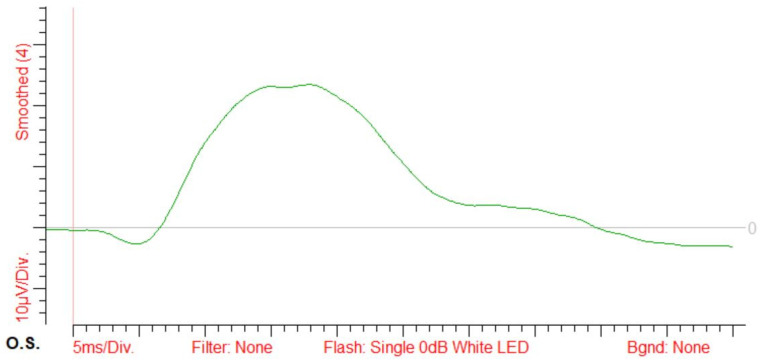
Representative electroretinogram waveform recorded at 0 dB under scotopic (dark-adapted) conditions in a collared scops owl (*Otus lettia*).

ERG recordings were successfully obtained from all individuals. Both the scotopic and photopic ERGs demonstrated distinguishable a- and b-waves. Representative ERG waveforms are shown in Figure 2. A distinct a-wave was observed as the initial negative deflection, followed by a prominent positive b-wave. The b-wave amplitude was greater than that of the a-wave, with a well-defined peak and a gradual return toward the baseline. Both the a- and b-wave components were identifiable, allowing us to measure the implicit times and amplitudes.

Because all the ERG parameters followed a Gaussian distribution, parametric statistical methods were applied. Results are presented as mean ± SD.

Under scotopic conditions at –20 dB, the a-wave amplitude was 1.78 ± 0.53 μV, with an implicit time of 37.83 ± 5.52 ms, whereas the b-wave amplitude was 41.59 ± 10.71 μV, with an implicit time of 100.88 ± 10.9 ms.

Under scotopic 0 dB conditions, the a-wave amplitude was 27.98 ± 5.9 μV with an implicit time of 27.64 ± 2.71 ms, and the b-wave amplitude was 175.51 ± 13.82 μV with an implicit time of 97.02 ± 7.01 ms. Under scotopic +5 dB conditions, the a-wave amplitude was 34.02 ± 9.08 μV with an implicit time of 29.53 ± 4.06 ms, and the b-wave amplitude was 239.1 ± 26.18 μV with an implicit time of 97.71 ± 8.32 ms.

Under photopic conditions, the a-wave amplitude was 2.88 ± 2.06 μV with an implicit time of 28.67 ± 2.77 ms, and the b-wave amplitude was 25.53 ± 10.61 μV with an implicit time of 77.78 ± 16.18 ms. A detailed summary of the ERG parameters is presented in Table 1.

## 4. Discussion

We successfully used flash ERG to characterize retinal function in free-living collared scops owls (*O. lettia*), providing the first set of species-specific baseline ERG parameters. A typical flash ERG waveform consists of an initial negative deflection (a-wave), followed by a subsequent positive deflection (b-wave). The amplitude and implicit time of these components must be determined to interpret the ERG recordings [16].

Multiple variables can influence ERG recordings, and their effects may be misinterpreted as changes attributable to retinal disease [16]. These variables can be broadly categorized into physiological and instrument-related factors. Physiological factors include the individual characteristics of the examined animal, such as species and breed, age, level of dark adaptation, retinal perfusion, and depth of anesthesia or sedation [17]. Equipment-related factors include the conductive properties of the electrodes, filter and amplifier settings, and stimulus-specific parameters such as light intensity and stimulus characteristics [18].

A crucial aspect of ERG interpretation involves the use of different stimulation and recording protocols, allowing the differentiation of responses arising from specific retinal cell types [7]. Dim stimuli under dark-adapted (scotopic) conditions are primarily used to elicit rod-mediated responses to comprehensively evaluate rod and cone photoreceptor functions, whereas bright stimuli under light-adapted (photopic) conditions predominantly evoke cone-driven activity. Moreover, a mixed rod–cone response obtained under dark-adapted conditions is routinely included in standard ERG protocols [9,11]. We used a series of protocols adapted from those established in previous studies to comprehensively and systematically assess the rod and cone functions.

The avian visual system is highly specialized and exhibits significant adaptations based on the lifestyle, habitat, and primary behavior of each species. Furthermore, these functional differences are reflected in ERG responses [19,20]. Retinal cone cells predominate over rod cells in diurnal raptors, supporting enhanced visual acuity under bright-light conditions. In contrast, nocturnal raptors have a higher proportion of rod cells than cone cells, which facilitates greater visual sensitivity in dim environments [21]. These anatomical differences in photoreceptor composition produce distinct ERG waveform characteristics between diurnal and nocturnal raptors [13]. Among avian orders, Strigiformes species exhibit a wide range of visual adaptations. Certain species display relatively photopic electroretinographic responses, whereas others are predominantly scotopic, reflecting varying degrees of nocturnality within this group [10].

In this study, ERG recordings in collared scops owls revealed stronger b-wave amplitudes under scotopic conditions, consistent with nocturnal visual adaptations. However, a direct comparison of ERG parameters across studies remains challenging because of a lack of standardized protocols. Variations in instrumentation, stimulus intensities, recording settings, and anesthetic regimens among laboratories can significantly influence amplitude and latency values, thereby limiting cross-study comparability [10]. Nevertheless, a comparison to other owl species, such as the barred owls, Eastern screech owls, and great horned owls, whose reported scotopic b-wave amplitudes range from approximately 148 to 481 µV, demonstrated that the collared scops owl in this study exhibited a range of 161 to 405 µV. Under photopic conditions, the previously mentioned species showed b-wave amplitudes ranging from 51 to 133 µV, whereas values in the collared scops owl were notably lower, ranging from 14.34 to 36.6 µV [11]. These findings suggest interspecies variations in retinal responses among Strigiformes.

In addition to the a- and b-waves, ERG can record oscillatory potentials (OPs), which are a series of small wavelets superimposed primarily on the ascending limb of the b-wave [7]. OPs are believed to originate from the inner retinal cells. Their numbers and morphologies vary among individuals and species [7]. They are most distinguishably observed under specific bandpass filter settings, which enhance their visibility by isolating the high-frequency components [10,13]. In the present study, OPs were not detected, which may be attributed either to their absence or underdevelopment in the collared scops owl or to the use of filter settings that were not optimized for OP detection.

The waveforms and parameters of ERG were influenced by the duration of both light and dark adaptations. Prolonged dark adaptation leads to a more accurate measurement of rod-mediated responses because it enables the retina to reach a more complete state of adaptation [22]. However, no standardized adaptation duration is currently available for avian ERG. The reported dark adaptation times vary substantially, ranging from 4 to 20 min, whereas light adaptation periods are most commonly set to 10 min [10,11,13]. In this study, dark and light adaptation times of 20 min and 10 min, respectively, were used. This extended dark adaptation period was selected to ensure optimal conditions for recording the rod-driven responses.

General anesthesia is commonly recommended for comprehensive ERG testing in veterinary patients because it facilitates proper fixation, ensures stable electrode positioning, and reduces motion artifacts and animal stress [23]. Inadequate sedation may result in excessive muscle activity, causing electrical interference in the ERG display, particularly after the b-wave peak [18]. Although ERG recordings can be performed in birds under manual restraint [11,24], anesthesia with isoflurane was used in the present study, in consideration of the wild origin of the subjects. This approach was intended to minimize potential stress and reduce the measurement variability associated with physical restraints.

ERG responses are widely known to be affected by general anesthesia, typically manifesting as reduced amplitudes and prolonged implicit times in both rod- and cone-driven activities. Numerous studies in dogs have demonstrated these anesthesia-associated alterations in ERG recordings [25,26,27]. In contrast, relevant literature on avian species is limited. A study in pigeons demonstrated the depressant effect of medetomidine on photopic ERG responses [28]. Although anesthetic agents can influence ERG recordings, interpretation is often compromised by the substantial motion artifacts commonly encountered in awake animals. When ERG data are acquired using identical protocols for preparation and anesthesia, the effects of anesthetics on the response amplitudes and implicit times are generally considered negligible [11]. Therefore, it is essential to standardize recording conditions to ensure reliable and comparable ERG results.

Environmental electromagnetic and electrostatic interference can complicate the initial setup of an ERG system by introducing noise artifacts that compromise signal fidelity [11]. Consistent with previous reports, we encountered similar sources of interference in our setup. Common contributors include unshielded power lines, switches, fluorescent lighting, computers, transformers, monitors, and network equipment. Adequate electromagnetic shielding should be implemented to obtain high-quality ERG recordings, and all unnecessary electrical equipment in the examination room should be turned off [7,18]. Although mitigating such interference can be time-consuming, it is crucial to ensure signal integrity. Clean ERG waveforms that require minimal post-acquisition correction substantially improve the reliability and diagnostic utility of examinations.

In avian clinical practice, ERG has been applied in different clinical scenarios, including the preoperative assessment of visual function before cataract surgery [4,29], evaluation of ocular consequences following traumatic injuries in wild raptors [3], detection of retinal dysfunction caused by nutritional deficiencies in quails [30], diagnosis of virus-induced blindness in African gray parrots [31], and assessment of potential retinal toxicity associated with specific pharmacological agents in penguins [32].

However, flash ERG primarily reflects the summed electrical response of the retina to light stimulation and may not accurately detect localized retinal abnormalities, particularly in cases of focal retinal injury [7]. This characteristic should be considered when interpreting ERG results, especially in wildlife undergoing pre-release assessments. Nevertheless, ERG provides an objective means of evaluating the overall retinal function and is valuable for identifying clinically significant retinal dysfunction. In this context, ERG should be considered a complementary diagnostic tool alongside ophthalmic examination, other imaging modalities, and relevant assessments of visual performance before release.

The limitations of this study include the relatively small sample size and the lack of detailed signalment information, particularly regarding the exact age of the owls. Certain collared scops owls admitted to wildlife rehabilitation centers for conservation purposes exhibited concurrent medical conditions, rendering them unsuitable for inclusion in the study and further limiting sample availability. Furthermore, all owls included in this study had previously been admitted to wildlife rehabilitation centers due to trauma or other medical conditions. Although all individuals had completed rehabilitation, were clinically stable, and were undergoing pre-release evaluations at the time of electroretinographic examination, and no ocular abnormalities were detected during comprehensive ophthalmic examinations, the potential effects of previous trauma on retinal function cannot be completely excluded. Therefore, a degree of selection bias may be present and should be considered when interpreting the reported parameters.

In addition, electroretinographic data from both eyes of each owl were included in the analyses. Because paired eyes from the same individual are not statistically independent observations, the potential for pseudoreplication cannot be entirely excluded. Therefore, the reported values should be interpreted as preliminary baseline electroretinographic parameters. Future studies involving larger sample sizes are warranted to further validate these findings.

Age-related changes in ERG have been reported in other species, including a reduction in response amplitude associated with an age-dependent decrease in photoreceptors [33]. Studies in pigeons have demonstrated significantly reduced ERG response slopes in older individuals than in younger individuals, suggesting age-related declines in visual acuity and photoreceptor function [34]. Accordingly, the ERG baseline values were established for different age groups [9].

However, precise age determination beyond adulthood is not feasible for free-living collared scops owls. All owls included in the present study were confirmed to be adults based on their plumage characteristics. Therefore, the ERG measurements reported here should be interpreted as adult baseline data, rather than age-stratified reference values.

In conclusion, this study presents the first comprehensive characterization of species-specific baseline ERG waveforms in collared scops owls (*Otus lettia*), incorporating both qualitative (morphological features) and quantitative (amplitude and implicit time) analyses. The functional characteristics of cone- and rod-mediated responses were independently evaluated using distinct recording protocols.

The baseline ERG parameters established in this study provide a critical foundation for the species-specific assessment of retinal function. The collared scops owl warrants specific attention not only because it is one of the most frequently rescued owl species in Taiwan, but also because it serves as a valuable model for understanding the retinal characteristics of nocturnal raptors.

The findings of this study provide a useful tool for clinical diagnosis and experimental research on avian retinal function and disease. In addition, this framework can be applied to other nocturnal raptors and avian species, thereby supporting future investigations in vision science and the development of therapeutic strategies for retinal disorders.

## Figures and Tables

**Table 1:** Electroretinographic parameters obtained under scotopic and photopic conditions in Collared scops owls (*Otus lettia*), including a- and b-wave amplitudes and their corresponding implicit times. Amplitudes are expressed in microvolts (µV), and implicit times in milliseconds (ms).

**Scotopic pure rod response under −20 dB stimulus**				
	a amplitude	a implicit	b amplitude	b implicit
Mean	1.78	37.83	41.59	100.88
SD	0.53	5.52	10.71	10.9
Minimum	1.25	30.5	32.22	89.25
Maximum	3.14	47.05	62.73	125
95% CI	1.45–2.12	34.32–41.33	34.79–48.4	93.95–107.8
	**Scotopic mixed rod-cone response under 0 dB stimulus**			
	a amplitude	a implicit	b amplitude	b implicit
Mean	27.98	27.64	175.51	97.02
SD	5.9	2.71	13.82	7.01
Minimum	20.97	24.85	152.33	88.75
Maximum	37.92	32.25	192.29	113.5
95% CI	24.23–31.73	25.92–29.36	166.73–184.3	92.57–101.47
**Scotopic mixed rod-cone response under +5 dB stimulus**				
	a amplitude	a implicit	b amplitude	b implicit
Mean	34.02	29.53	239.1	97.71
SD	9.08	4.06	26.18	8.32
Minimum	5.69	24.25	208.42	90
Maximum	73.06	38.5	289.12	115
95% CI	28.89–39.16	26.95–32.11	222.46–255.73	92.42–102.99
	**Photopic pure cone response under 0 dB stimulus**			
	a amplitude	a implicit	b amplitude	b implicit
Mean	2.88	28.67	25.53	77.78
SD	2.06	2.77	10.61	16.18
Minimum	0.83	24.5	7.92	56.5
Maximum	6.81	34.25	41.53	104.25
95% CI	1.57–4.19	26.91–30.43	18.79–32.28	67.49–88.06

Data are represented as mean, standard deviation (SD), minimum, maximum, and 95% confidence intervals (CI).

## Data Availability

The original contributions presented in this study are included in the article. Further inquiries can be directed to the corresponding author.

## Abbreviations

The following abbreviations are used in this manuscript:

ERG	Electroretinography
SD	Standard deviation
CI	Confidence interval
OPs	Oscillatory potentials

## References

[1] O’Malley B. (2005). Clinical Anatomy and Physiology of Exotic Species Structure and Function of Mammals, Birds, Reptiles, and Amphibians.

[2] Keenan A.V., Oster S., McMullen R.J., Shaw G.C., Dubielzig R.R., Teixeira L.B.C., Bellah J.R., Moore P.A., Boveland S.D. (2022). Clinical and pathologic evaluation of chorioretinal lesions in wild owl species. Vet. Ophthalmol..

[3] Seruca C., Molina-López R., Peña T., Leiva M. (2012). Ocular consequences of blunt trauma in two species of nocturnal raptors (*Athene noctua* and *Otus scops*). Vet. Ophthalmol..

[4] Carter R.T., Murphy C.J., Stuhr C.M., Diehl K.A. (2007). Bilateral phacoemulsification and intraocular lens implantation in a great horned owl. J. Am. Vet. Med. Assoc..

[5] Rainwater K.L., Sykes J.M., Sapienza J.S. (2015). Retrospective investigation of cataract management in avian species in a zoologic collection. J. Zoo Wildl. Med..

[6] Pauli A., Klauss G., Diehl K., Redig P. (2007). Clinical techniques: Considerations for release of raptors with ocular disease. J. Exot. Pet. Med..

[7] Gelatt K.N. (2021). Veterinary Ophthalmology.

[8] Maggs D.J., Miller P.E., Ofri R., Maggs D.J., Miller P.E., Ofri R. (2018). Slatter’s fundamentals of veterinary ophthalmology. Fundamentals of Veterinary Ophthalmology.

[9] Ekesten B., Komáromy A.M., Ofri R., Petersen-Jones S.M., Narfström K. (2013). Guidelines for clinical electroretinography in the dog: 2012 update. Doc. Ophthalmol..

[10] Hendrix D.V.H., Sims M.H. (2004). Electroretinography in the Hispaniolan Amazon parrot (*Amazona ventralis*). J. Avian Med. Surg..

[11] Labelle A.L., Whittington J.K., Breaux C.B., Labelle P., Mitchell M.A., Zarfoss M.K., Schmidt S.A., Hamor R.E. (2012). Clinical utility of a complete diagnostic protocol for the ocular evaluation of free-living raptors. Vet. Ophthalmol..

[12] Porciatti V., Fontanesi G., Bagnoli P. (1989). The electroretinogram of the little owl (*Athene noctua*). Vis. Res..

[13] Kuhn S.E., Hendrix D.V.H., Sims M.H., Ward D.A., Jones M.P., Baine K.H. (2014). Flash Electroretinography in the bald eagle (*Haliaeetus leucocephalus*). J. Zoo Wildl. Med..

[14] Chan F.-T., Lin P.-I., Chang G.-R., Wang H.-C., Hsu T.-H. (2012). Hematocrit and plasma chemistry values in adult Collared scops owls (*Otus lettia*) and crested serpent eagles (*Spilornis cheela hoya*). J. Vet. Med. Sci..

[15] Chiu Y.S., Chie C.H., Colitz C.M., Huang W.H., Yang Y.W., Lin C.T. (2025). Retinal morphological characterization of Collared scops owl (*Otus lettia*) using optical coherence tomography and histological techniques. Vet. Ophthalmol..

[16] Drazek M., Lew M., Lew S., Pomianowski A. (2014). Electroretinography in dogs: A review. Vet. Med..

[17] Mentzer A.E., Eifler D.M., Montiani-Ferreira F., Tuntivanich N., Forcier J.Q., Petersen-Jones S.M. (2005). Influence of recording electrode type and reference electrode position on the canine electroretinogram. Doc. Ophthalmol..

[18] Komáromy A.M., Brooks D.E., Dawson W.W., Källberg M.E., Ollivier F.J., Ofri R. (2002). Technical issues in electrodiagnostic recording. Vet. Ophthalmol..

[19] Rojas L.M., McNeil R., Cabana T., Lachapelle P. (1999). Diurnal and nocturnal visual capabilities in shorebirds as a function of their feeding strategies. Brain Behav. Evol..

[20] Rojas L.M., McNeil R., Cabana T., Lachapelle P. (1999). Behavioral, morphological and physiological correlates of diurnal and nocturnal vision in selected wading bird species. Brain Behav. Evol..

[21] Ruggeri M., Major J.J.C., McKeown C., Knighton R.W., Puliafito C.A., Jiao S. (2010). Retinal structure of birds of prey revealed by ultra-high resolution spectral-domain optical coherence tomography. Investig. Ophth Vis. Sci..

[22] Bach M., Meroni C., Heinrich S.P. (2020). ERG shrinks by 10% when reducing dark adaptation time to 10 min, but only for weak flashes. Doc. Ophthalmol..

[23] Narfström K., Ekesten B., Rosolen S.G., Spiess B.M., Percicot C.L., Ofri R. (2002). Guidelines for clinical electroretinography in the dog. Doc. Ophthalmol..

[24] Ookawa T. (1971). Effect of light and dark adaptation on the ERG of unanesthetized and anesthetized chicks. Poult. Sci..

[25] Freeman K.S., Good K.L., Kass P.H., Park S.A., Nestorowicz N., Ofri R. (2013). Effects of chemical restraint on electroretinograms recorded sequentially in awake, sedated, and anesthetized dogs. Am. J. Vet. Res..

[26] Rosolen S.G., Rigaudiere F., Lachapelle P. (2002). A practical method to obtain reproducible binocular electroretinograms in dogs. Doc. Ophthalmol..

[27] Tanskanen P., Kylmä T., Kommonen B., Karhunen U. (1996). Propofol influences the electroretinogram to a lesser degree than thiopentone. Acta Anaesthesiol. Scand..

[28] Susanti L., Kang S., Park S., Park E., Park Y., Kim B., Kim S., Seo K. (2019). Effect of three different sedatives on electroretinography recordings in domestic pigeons (*Columba livia*). J. Avian Med. Surg..

[29] Sigmund A.B., Jones M.P., Ward D.A., Hendrix D.V.H. (2019). Long-term outcome of phacoemulsification in raptors—A retrospective study (1999–2014). Vet. Ophthalmol..

[30] Endo K., Itoh N., Maehara S., Shinozaki A., Imagawa T., Uehara M., Mizuno N., Sasaki S., Hiraga T., Teraoka H. (2008). Functional disorder of the retina in manganese-deficient Japanese quail revealed by electroretinography using a contact lens electrode with built-in light source. J. Vet. Med. Sci..

[31] Steinmetz A., Pees M., Schmidt V., Weber M., Krautwald-Junghanns M.E., Oechtering G. (2008). Blindness as a sign of proventricular dilatation disease in a grey parrot (*Psittacus erithacus erithacus*). J. Small Anim. Pract..

[32] Ross M., Avni-Magen N., Pe’er O., Berkowitz A., Ofri R. (2021). Treatment with chloroquine is retinotoxic in captive African penguins (*Speniscus demersus*). Attenuation and recovery of electroretinographic responses. Vet. Ophthalmol..

[33] Dorey C.K., Wu G., Ebenstein D., Garsd A., Weiter J. (1989). Cell loss in the aging retina. Relationship to lipofuscin accumulation and macular degeneration. Investig. Ophth Vis. Sci..

[34] Porciatti V., Hodos W., Signorini G., Bramanti F. (1991). Electroretinographic changes in aged pigeons. Vis. Res..

